# Confirming mental health care in acute psychiatric wards, as narrated by persons experiencing psychotic illness: an interview study

**DOI:** 10.1186/s12912-016-0126-x

**Published:** 2016-01-13

**Authors:** Karina Sebergsen, Astrid Norberg, Anne-Grethe Talseth

**Affiliations:** Department of Health and Care Sciences, Faculty of Health Sciences, UiT The Arctic University of Norway, N-9037 Tromsø, Norway; Division of Mental Health and Substance Abuse, University Hospital of North Norway, Mailbox 6124, N-9291 Tromsø, Norway; Department of Nursing, Umeå University, SE-90187 Umeå, Sweden; Palliative Research Center, Ersta Sköndal University College, SE-10061 Stockholm, Sweden

**Keywords:** Acute psychotic illness, Confirmation, Mental health nursing care, Narrative, Qualitative research

## Abstract

**Background:**

It is important that mental health nurses meet the safety, security and care needs of persons suffering from psychotic illness to enhance these persons’ likelihood of feeling better during their time in acute psychiatric wards. Certain persons in care describe nurses’ mental health care as positive, whereas others report negative experiences and express a desire for improvements. There is limited research on how persons with psychotic illness experience nurses’ mental health care acts and how such acts help these persons feel better. Therefore, the aim of this study was to explore, describe and understand how the mental health nurses in acute psychiatric wards provide care that helps persons who experienced psychotic illness to feel better, as narrated by these persons.

**Method:**

This study had a qualitative design; 12 persons participated in qualitative interviews. The interviews were transcribed, content analysed and interpreted using Martin Buber’s concept of confirmation.

**Results:**

The results of this study show three categories of confirming mental health care that describe what helped the participants to feel better step-by-step: first, being confirmed as a person experiencing psychotic illness in need of endurance; second, being confirmed as a person experiencing psychotic illness in need of decreased psychotic symptoms; and third, being confirmed as a person experiencing psychotic illness in need of support in daily life. The underlying meaning of the categories and of subcategories were interpreted and formulated as the theme; confirming mental health care to persons experiencing psychotic illness.

**Conclusion:**

Confirming mental health care acts seem to help persons to feel better in a step-wise manner during psychotic illness. Nurses’ openness and sensitivity to the changing care needs of persons who suffer from psychotic illness create moments of confirmation within caring acts that concretely help the persons to feel better and that may enhance their health. The results show the importance of taking the experiential knowledge of persons who have experienced psychotic illness seriously to develop and increase the quality of mental health care in acute psychiatric wards.

## Background

Psychosis affects about 1 % of the population worldwide once in a life time. The incidence of new cases of psychosis is estimated to 15–20 per 100 000 inhabitants a year [1, 2]. This means 750–1000 new cases of persons suffering from psychosis in Norway a year, and in the Nordic countries the total of 3900–5200 new cases. Most persons recover from psychosis, although experience vulnerabilities to new phases of psychosis, and/or some persons have long-lasting psychotic illness [1]. In the last decade, there has been a new emphasis in the global community on developing and offering the best possible mental health care to prevent psychosis, and to reduce persons suffering from psychotic illness and to support their daily life [3].

Psychosis is defined by certain symptoms, such as hallucinations, delusions and disturbed behaviour, and refers to psychotic illnesses [4]. Persons experiencing psychotic illness describe a changeable phenomenon with risks of developing acute phases of psychosis [1, 2]. The acute phase of psychotic illness can be characterized by increased distress and psychotic symptoms that include distortions in emotions, thinking, perceptions, sense of self and behaviour [1, 2]. Being acute psychotic ill is metaphorically described as being adrift from one’s own body and self, from other people and from the environment (cf. [5]). Persons may express severe mental distress and suffering from psychotic illness as complex and challenging mental health care needs [6]. Clinical mental health care guidelines recommend a range of clinical approaches to the various phases of psychosis within different care settings and specific mental health care adjusted for each person [1, 2].

In most Western countries, mental health care for persons experiencing psychotic illness is primarily offered in the community and secondarily in acute psychiatric wards in hospitals. Mental health professionals in acute psychiatric wards seek to diagnose, treat and provide intensive care for persons to reduce their psychotic symptoms and enhance their health [7]. Research studies have reported growing evidence of the potential to prevent psychosis and/or decrease psychotic symptoms to improve persons’ health with treatment and care in different care settings [4, 8]. Researchers have recommended that mental health nursing care should be person-centred, meaning that care acts should be provided within the interpersonal relationship between the nurse and the person in care, based on how the person in care understands his/her situation and needs and in accordance with what the nurse understands is the person’s care needs [5, 9].

The main task for mental health nurses in acute psychiatric wards is to meet the person’s emergent needs for safety and security and his/her physical and mental needs while he/she experiences psychotic illness [10, 11]. This requires trained mental health nurses with specific knowledge about and skills in mental health care for the person, social interaction and communication [12–14]. The interpersonal interaction between the nurse and the person in care is considered as a cornerstone of mental health care [15]. It is important to be aware that the efficiency demands in acute psychiatric wards may prevent nurses from interacting with persons in their care [16, 17]. To better understand mental health care acts, we reviewed the literature on how persons with psychosis experience and describe the mental health care they receive in acute psychiatric wards as a help to feel better. Mental health nurses’ responsibility is to help and support the person who experiences psychotic illness to “get going again” and “feel better” [9]. The goal of mental health nurses care is what is possible for the person in care. This can be “to feel better” which is the expression nurses relate to when providing mental health care (cf. [18]).

Some studies report positive experiences in acute mental health care. One ethnographic study described that nurses’ regulation and control of the admitted persons’ behaviour were intended to meet their needs for safety and security during acute mental illness [19]. Interview studies have highlighted that persons receiving acute mental health care experience the acute psychiatric ward as a refuge from self-destructiveness [20] and as a safe place [21] that frees them from daily chaos and stress [22]. Furthermore, persons described nurses as trustworthy and fair [21, 23] and reported that the nurses confirmed them as persons [24]. The relationships with nurses increased the perceived quality of care to persons in care [25], and they appreciated that nurses were available to them on the ward [26]. The quality of life for persons increased when nurses supported their physical health, and helped them to cope with symptoms and daily life problems, and when nurses supported contact with their families [27]. The persons in care were helped when their psychosis were alleviated by nurses who tried to understand their vulnerability and helped them to be empowered [20]. The interpersonal relationship with nurses was described as important in improving the mental health to persons receiving mental health care [28, 29], and being involved in medical treatment and care decisions helped them feel in control [30–32].

Other studies have described persons’ experiences with acute mental health care in negative terms. In two different ethnographic studies, researchers reported that mental health care lacks interaction between nurses and persons in care because nurses spend most of their time working at nursing stations [33, 34]. These results are similar to those of two review studies [35, 36]. Other persons who experience psychotic illness reported that staying in an acute ward was meaningless and was similar to being in a prison because of the sense of intimidation and control and the long waits to see nurses. They doubted the effectiveness of the care they received during their acute psychotic illness [37]. Still others described dissatisfaction with their care because of restrictions such as compulsion and confinement, which increased their mental distress [38, 39], and because they felt they were humiliated and disrespected [40]. A survey study revealed that a controlling, angry and aggressive atmosphere on the ward decreased the quality of care provided [41].

Studies have also shown that persons in acute psychiatric wards want to interact with nurses [25] who are trained and understanding and have good social skills; furthermore, care recipients want to be perceived as ill persons who need care [23, 40]. Some involuntarily admitted persons desired more communication with the staff about their care [39]. Persons receiving care wanted to discuss and negotiate with nurses how their care could be provided [42], desired to be engaged in their treatment [23], and longed for deeper connections with nurses to share their inner world [20].

The reviewed studies described that some persons experienced mental health care as positive and helpful when their care needs were met within the context of a positive interpersonal relationship with nurses, such as being helped when protected from vulnerabilities and empowered during psychotic illness [20]. However, mental health care was also described in negative terms when persons experienced a lack of interpersonal relationships and interaction with nurses and when the care included confinement and restrictions. Persons in care in psychiatric wards wanted to be acknowledged and respected as a person by the nurse. There is limited research on how persons with psychotic illness experience care acts in their relationships with nurses, and whether that care helps them to feel better. Nursing care comprises two aspects: one is the issue/matter act and the other is the interpersonal relationship between the nurse and the person in care that strengthens the quality of the care act (cf. [5, 43, 44]). These two aspects of nursing are interwoven. Mental health nursing care in acute psychiatric wards is intended to provide a safe haven through interpersonal relationships between the nurses and the persons in care by addressing the latter’s care needs and supporting their recourses. Within this perspective and to the little known about how nurses’ mental health care acts help persons to feel better, we designed a study to assess the care experiences of persons who suffer from psychotic illness. The aim of this study is to explore and describe how the mental health care provided by nurses was experienced as help to feel better, as narrated by persons with psychotic illness in acute psychiatric wards.

We employed Martin Buber’s [45] concept of confirmation to understand the mental health care act as a confirming act. According to Buber [45], confirmation happens in events of confirming acts; it is not the event itself, but rather what happens between people. Confirmation happens when one person apprehends the other as a different and unique person, and meets him/her as an independent other able to enter relationships in which they confirm each other as persons. Confirmation is fundamental to the formation of a person’s self and identity [45]. The concept of confirmation has been used in mental health nursing research to understand the relationships between the person in care and the nurse [24, 46].

## Methods

To address the aim of this study, we used a qualitative, explorative and descriptive study design. Qualitative interviews we found suitable for assessing persons’ narrated experiences of psychotic illness were used [47]). To analyse the interview texts, we chose a qualitative content analysis that allows description of the manifest content of the data, interpretation of the underlying meaning of subcategories and categories, and formulation of a theme [48]. Through the qualitative content analysis, the multifaceted and sensitive phenomenon of nursing care can be uncovered, explored and described (cf. [49]).

### Ethics

The Regional Committee for Medical and Health Research Ethics (2012/1319) approved the study. Key research ethical principles were followed: the participants received written and oral information about the study and on the voluntary nature of participation. They signed an informed consent form prior to participation; confidentiality and the anonymous presentation of the results were guaranteed; and the participants were assured of their right to withdraw from the study without any explanation at any time before the analysis with no consequences for their treatment or care. The researchers were aware of the vulnerability of persons who are hospitalized for psychotic illnesses, possible limitations in their ability to understand and sign the informed consent form, and the risk that they may feel coerced to participate (cf. [50, 51]). According to the study procedure, the local project contact person on each ward contacted the participants after the interviews. This contact person ensured that the primary nurse followed up the participants if the interview had evoked bad memories that they strived to cope with.

### Setting

For the past 10–15 years in Norway, mental health care has been offered in acute psychiatric wards of hospitals to persons experiencing acute phases of psychotic illness when community mental health care is insufficient. The setting for this study was four acute psychiatric wards at two general hospitals. The hospitals located in mid-sized cities are responsible for providing acute mental health services to large geographical areas, which implies relatively long distances between the hospitals and the admitted persons’ homes. Each 10- to 12-bed ward provides short-term treatment and care (for a mean of 10 days) to women and men experiencing acute mental illness. Each ward reported admitting approximately 400 people per year. The nursing staff members are mental health nurses, nursing assistants and registered nurses who are educated and trained in acute mental health care. Together with the persons in care and, often, the latter’s family members, the multi-professional team on the ward is involved in developing a mental health care plan for each person in care on the ward.

### Recruitment procedure

The participants were purposively recruited from among admitted persons on four acute wards during a five-month period and were selected based on voluntary or involuntary admission, psychotic illness, and their willingness to narrate their experiences of mental health care in acute psychiatric wards. The chief physician and the responsible nurse on the ward selected persons in care to be asked about participation after the acute phase of psychosis. In cooperation with the person selected; the physician and nurse ensured that he/she understood what it means to participate in the research; including narrating his/her experiences of psychotic illness (cf. [51]). Those 20 persons selected for recruitment received written and oral information from a local project contact person at each hospital who did not work in mental health care on the ward. Fourteen persons provided informed consent for participation; however, two persons discharged from hospital before an interview could be arranged. The number of participants and the variation in the sample appeared sufficient to describe the nuances and variations in experience and were small enough to allow a thorough analysis of the data (cf. [48]).

### Participants

Eight women and four men aged 18 to 64 years participated. Eight of the participants were involuntarily admitted, and four were voluntarily admitted according to the Norwegian Mental Health Act [52]. The participants reported they had been diagnosed with psychosis during an earlier hospital stay. They described their number of stays in acute wards as ranging from two to approximately 40. This period of admission to the acute psychiatric wards was related to a phase of psychotic illness. The stays during which the interviews were conducted lasted from one to approximately eight weeks. All of the participants reported having contact with their family members. Eight of them lived together with partners or other family members, and four lived alone in their own flat or house.

### Interviews

Only the first author (KS) and the interviewee were present during the interview, which was conducted in the hospital in a quiet room outside the ward. Before the interview began, KS introduced herself, provided information about the research study and discussed everyday matters, such as place of residence. KS then repeated the information about the study, the interview, and audio recording and began a conversation about how the participants experienced the interview situation. The qualitative interview [47] began with an opening question inviting the participants to speak freely about and narrate the mental health care they received. Additional and exploratory questions followed, such as “Please describe what happened during your care”; “Please tell me what you felt, thought, wished for during your care”; “How did you experience that the mental health care helped you feel better?” An interview guide was used to ensure that various aspects of the topic were covered. The interviews lasted 50–90 minutes, not counting the time taken for breaks, and were audio recorded and transcribed verbatim by KS. When the twelfth interview did not reveal any new information on the study topic, no further interviews were conducted (cf. [53]).

### Analysis

A qualitative content analysis inspired by Graneheim & Lundman [48] was used in a step-wise manner:

- It should be noted that the interview text included descriptions of mental health care that the participants regarded as either helping or not helping them feel better. In the present study, the interview text from all interviews that describes the mental health care that helped the participants feel better constitutes the unit of analysis. This interview text was read and reread in an open-minded manner to gain a sense of the whole according to the aim of the study. In this reading, we recognized elements of person-centred mental health care (cf. [5]) in the interview text that described care that responded to each participant’s personal care needs when psychotically ill. These descriptions reminded us of Martin Buber’s [45] concept of confirmation.
- The interview text was divided into meaning units consisting of one or more sentences or a paragraph containing one meaning that described helping mental health care.
- Each meaning unit was condensed and labelled with codes via reflecting upon the following questions: What does the text describe as helping mental health care? How was the care helping feel better? To whom did the participants relate during care and how?
- The research team compared, contrasted and explored the commonalities and differences between the coded meaning units, which then were sorted and organized into subcategories and categories and validated by relating the subcategories. We then searched for uniformities to reduce the number of categories.
- The categories and subcategories were labelled with regard to confirmation (Tables 1 and 2).
- The underlying meaning of the subcategories and categories was interpreted in the context of the whole of all interviews, answering the question of how the participants experienced mental health care that helped them feel better. The interpretation of the underlying meaning of the subcategories and categories constructed from the total interview text was formulated as a theme: *Confirming mental health care to persons experiencing psychotic illness.*

**Table 1:** Example of the analysis process

Meaning unit	Condensed meaning unit	Subcategory	Category
The physician asked repeatedly if I accepted the treatment, as if to persuade me and I got stressed. (..) I said no, because I thought this treatment could be a danger to my body. When the nurse heard this, she said, “This is not the way it should be – I will talk with the Physician A”. Then, A stopped (…)	The participant talks about being supported and respected by the nurse and the physician.	Being in relationships with nurses and physicians	Being confirmed as a person experiencing psychotic illness in need of decreased psychotic symptoms
We talk about the psychosis and how it was when I injured myself. We talk about what happened before and put labels on a board to look at it. In this way, it gets easier for me and for them to understand (4).	The participant described how she and the nurse/physician can discuss symptoms to understand how she experiences them.	Being in discussion with nurses and physicians	

The example of the analysis may seem linear from meaning units to categories. Note that in the analysis, each category implies several subcategories, and each subcategory implies several condensed meaning units

**Table 2:** Interpreted theme, categories and subcategories

Theme	Confirming mental health care to persons experiencing psychotic illness		
Categories	Being confirmed as a person experiencing acute psychotic illness in need of endurance	Being confirmed as a person experiencing psychotic illness in need of decreased psychotic symptoms	Being confirmed as a person experiencing psychotic illness in need of support in daily life
Subcategories	Being understood by nurses as a person experiencing critical psychotic illness	Being understood by nurses and physicians as a person experiencing psychotic symptoms	Being understood by nurses as a vulnerable person feeling better
	Being in peaceful communication with nurses	Being in discussion with nurses and physicians	Being in dialogue with nurses
	Being in an unconditional relationship with nurses	Being in a relationship with nurses and physicians	Being in a partnership with nurses

## Results

**Figure 1:**
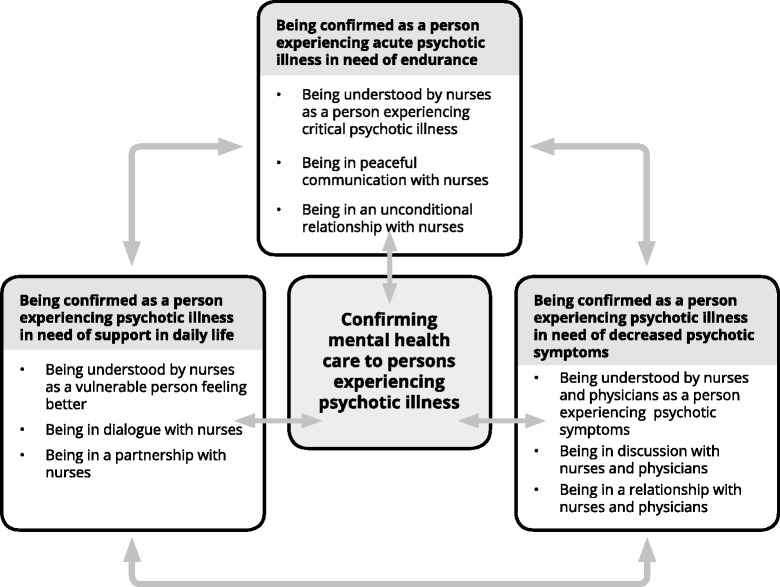
Confirming mental health care to persons experiencing psychotic illness. Confirming mental health care to persons experiencing psychotic illness links the three categories of nurses confirming acts: being confirmed as a person experiencing acute psychotic illness in need of endurance; being confirmed as a person experiencing psychotic illness in need of decreased psychotic symptoms; being confirmed as a person experiencing psychotic illness in need of support in daily life. These confirming acts help the person in a step-by-step movement towards to feel better. The movement of confirming care acts can also go back and forward, as well move into new circles of confirming mental health care

The results present how the participants in this study experienced the mental health care in acute psychiatric wards provided by nurses, helped them feeling better while they experienced psychotic illness. The theme, “confirming mental health care to persons experiencing psychotic illness”, links subcategories and categories together. Each of the three categories describes how the participants were approached by nurses and describe the nurses’ confirming mental health care acts. These confirming acts help the participants in a step-by-step movement towards to feel better. The movement of confirming care acts can also go back and forward, as well move into new circles of confirming mental health care. A schematic presentation of the results is illustrated in Fig. 1. The following presentation of the results consists of the theme, categories and subcategories and includes quotations from the interviews, numbered from 1–12, to verify the results.

### Confirming mental health care to persons experiencing psychotic illness

The results regarding confirming mental health care that involved the participants and the nurses in various mental health care acts during the participants’ acute psychotic illness illustrate how they were helped to feel better. The participants described that the mental health care helped them and confirmed their personal changing needs during their psychotic illness and that the confirming way in which the nurses provided mental health care helped them to feel better. The confirmation that occurred within the various confirming mental health care acts helped the participants feel better in a step-by-step manner during their psychotic illness by helping them become aware of their own care needs, increasing their self-respect, and strengthening their self-esteem as a person who matters to others. The various ways of being confirmed within mental health care acts are described further below.

### Being confirmed as a person experiencing acute psychotic illness in need of endurance

The participants described their experience during the first phase of psychotic illness as critical. The nurses’ unconditional mental health care confirmed their immediate need for care and helped them to endure the critical phase of psychotic illness. These confirming mental health care acts increased the participants’ awareness of their own needs.

#### Being understood by nurses as a person experiencing critical psychotic illness

The participants described that it was difficult to articulate their suffering and their care needs to nurses during this phase of psychotic illness. However, they knew that the nurses understood their suffering based on how the nurses’ mental health care acts addressed their immediate needs for safety, security and care. They described how the nurses remained with them, consoled them and assured them that their acute psychosis would pass. Without questioning their needs, the nurses provided nursing care, offered them medication and care to help them rest and sleep and to decrease their suffering, and limited their behaviour to prevent them from harming themselves or others. Being understood and receiving confirming mental health care in this way helped the participants endure the critical phase of their psychotic illness and helped them become aware of their own needs, as one participant articulated:

I was desperately anxious about Nurse A sitting there as if blocking the door, and I wanted out of the wardroom. Then, Nurse B offered to be with me. He did his best to ensure that everything would be OK. (…) He listened to how it really was for me and tried to understand me as well as possible (…), and maybe I only needed to be cared about (8).

#### Being in peaceful communication with nurses

The participants expressed that while they were critically psychotically ill, they were intensely mentally distressed and afraid of losing control over their behaviour. The nurses’ peaceful non-verbal and verbal communication helped them feel safer and find some peace, and the participants described how the nurses sat quietly with them and read the newspaper or completed their chores in the wardroom. The nurses used few words and did not demand any conversation. Furthermore, peacefulness was communicated through the nurses’ silent movement, silence, compassionate words and/or the use of a sensitive voice when providing care, limiting participants’ behaviour and/or explaining to the participants why care was needed. One participant illustrated this peaceful communication when describing how Nurse C approached her:

When I was psychotic, (…) I sometimes needed limitation. Nurse C, whom I have met several times, limited me in a very good and helpful manner. He used his body to stop me, and he murmured quietly, such as “hum-hum”, and did not argue with me… It made me feel safe (11).

#### Being in an unconditional relationship with nurses

The participants recalled being anxious during the critical phase of psychotic illness; they were anxious about being left alone and about being with people, and sometimes they rejected the nurses. The participants described the nurses who stayed with them, despite the participants’ hostility, as “special”. They described how these nurses acted with sensitivity to them and respected their personal space when providing care, such as when the participants were held and controlled and/or given personal care. They expressed that the nurses were physically close to their bodies without intruding, and one participant described the nurse’s sensitivity by making small, nearly imperceptible movements with her fingers. The participants expressed their gratitude for and emotional connection with the nurses who unconditionally provided mental health care in accordance with participants’ needs and sensitively balanced the participants’ needs for distance and closeness during nursing care in a way that felt safe and good. One participant described how Nurse D approached her:

It is very difficult to ask for help when you are this ill. Nurse D came and knocked on the door to the wardroom and asked to come in; I said, “No”. She said she would come back in five minutes. She returned, came in, and sat down and held her arms around me for a long time without saying much. I cried and told her I was about to harm myself. Her actions stopped me from harming myself (4).

### Being confirmed as a person experiencing psychotic illness in need of decreased psychotic symptoms

The participants described their suffering from psychotic symptoms. The nurses’ and physicians’ mental health care helped them decrease their suffering, and these confirming mental health care acts were expressed in terms of strengthening the participants’ self-respect and their relationships with nurses and physicians.

#### Being understood by nurses and physicians as a person experiencing psychotic symptoms

The participants stated that both nurses and physicians understood that their psychotic symptoms affected their daily activity and well-being. This understanding led the participants to trust the nurses’ and physicians’ competence and their desire for the participants to be well, and the participants accepted the invitation to be involved in planning their treatment and care. They described that the physicians were engaged in understanding how they experienced symptoms and the medical treatment they were offered and strove to determine the medication and doses that would effectively decrease their symptoms. The participants expressed that it was important to them that the nurses involved in planning their treatment and mental health care focused on and understood that they were afraid of the antipsychotic medications and that the medications could make them uncomfortable and/or hurt them. The participants appreciated the nurses’ support as they expressed these fears and experiences. One participant described the nurses’ understanding approach as follows:

Nurse F asked me about how the medication was for me, and I said: “It helps to reduce the big ups and downs, but has changed my daily life into grey days”. When hearing this, the physician suggested finding a new medication, and we sat together – me, the physician and the nurse, and I asked about everything, and they explained. They understood how important it was for me to feel better (2).

#### Being in discussion with nurses and physicians

The participants expressed that the nurses and physicians heard them during discussions about medication, and this made participants feel more in control. The participants raised questions and voiced opinions and desires, and they agreed, disagreed and negotiated with the physicians until they reached agreements and/or solutions about their treatment. The participants appreciated that nurses and physicians considered their accounts of the medications’ effects important and allowed disagreement. This increased the participants’ self-respect and encouraged them to express themselves and listen to the nurses’ and physicians’ opinions about medications. One participant described a discussion with the nurse and the psychiatrist as follows:

Regarding antipsychotic and sleeping medications, we had an uncompromising disagreement, but we agreed about our disagreement. However, I was heard, and they respected my opinion, and the psychiatrist took the initiative and offered me sleeping medication (11).

#### Being in a relationship with nurses and physicians

The nurses’ and physicians’ engagement in finding common solutions for the participants’ treatment and mental health care strengthened the participants’ relationships with the nurses and physicians and encouraged them to trust that the nurses/physicians cared about them. They appreciated that the nurses and physicians took the time to listen to them and respected their knowledge and opinions about medications, and they appreciated informal talks that allowed all of parties to get to know one another. They stated that their relationships with the nurses and physicians were different; they and the nurses knew one another more personally because they spent more time together and had more personal conversations. They valued their relationships with the nurses and the support that the nurses offered when they disagreed with the physicians about their treatment. The participants believed that the nurses’ support made a difference, as one participant described:

The physician repeatedly asked if I accepted the treatment, as if to persuade me, and I got stressed. (…) I said no, because I believed this treatment could be a danger to my body. When the nurse heard this, she said, “This is not the way it should be – I will talk with Physician A”. Then, A stopped. I think the nurse’s intervention made the physician stop (3).

### Being confirmed as a person experiencing psychotic illness in need of support in daily life

After being on the ward for some time, the participants felt better and were able to concentrate again. They described that at that point of time, the nurses involved them in plans for their discharge and further support in daily life at home. This approach confirmed the participants and strengthened their self-esteem.

#### Being understood by nurses as a vulnerable person feeling better

The participants noted that they were involved in planning their discharge and the support that they and their family members would need at home. They articulated that the nurse responsible for planning the discharge understood their vulnerability, their concerns about becoming psychotically ill again and their limitations at home due to the risk of psychosis. Together with the nurse and the team on the ward, the participants’ family members and local mental health professionals were invited to participate in planning the further support. The participants described how the nurses understood their vulnerability to stigmatization and the nurses’ trusted their competence to represent their needs. The following participant statement captures this experience:

Yesterday, my nurse and my team on ward invited the local treatment team to a meeting, and I suggested one important and useful theme to be discussed: the stigmatization of me at home (11).

#### Being in dialogue with nurses

Regarding meetings with family members and local mental health professionals, which were arranged at the hospital or as phone/video conferences, the participants described that the nurse asked what they wished to talk about. The participants expressed their worries about future support and medication, and their family members asked questions about what to do if the participants needed acute help. They also discussed disagreements between family members and participants that could occur at home, and the participants found it easier to discuss this topic when the nurses and the team were present and supported the conversation. The participants were emotionally affected when family members expressed their love for them despite their concerns. These dialogues reminded the participants that they and their family members mattered to one another, and they viewed their family members in a new way, as one participant described:

When I heard my mother speak, I heard she was afraid of losing me or not knowing where I was. Not for control, but to know. (…) I understand more of my mother’s worries about me now (4).

#### Being in a partnership with nurses

Cooperating with the nurse on the ward to plan the discharge and find solutions for mental health care at home strengthened the participants’ relationship with the nurse as a partner. The participants described how they used their knowledge of their own mental illness and care needs in the cooperation with the nurse and how the nurse used his/her nursing knowledge about psychosis and planning the discharge. Some participants expressed feeling ashamed about their illness and dependence and appreciated the nurses’ sensitive behaviour, honesty and acceptance of their care needs. The participants cared about the nurses who approached them in this manner, as one participant described:

Nurse G and me have known each other for years during several admissions. She was the one who helped me receive benefits and keep in contact with my children. She supports me by trusting my needs and helps me figuring out about further treatment. I am still weak and need support. We are a kind of a team (9).

## Discussion

Our results indicate that it is important for persons experiencing psychotic illness to receive confirming mental health care to help them to feel better. Confirming mental health care includes three approaches that address and confirm participants’ personal changing needs for mental health care during psychotic illness. We do not know whether the participants described exactly what happened, but we know that they described their experiences of confirming mental health care. The participants’ narratives help uncover differences in confirming approaches and in how the confirmation occurred during interactions between the nurses and the participants in mental health care acts. We seek to understand how confirming mental health care were received and how the confirming mental health care acts helped persons with psychotic illness feel better, step-by-step, in acute psychiatric wards. The results are discussed with respect to previous research and Buber’s [45] concept of confirmation.

The nurses’ confirming mental health care acts addressed the participants’ immediate needs for safety, security and care to help them to endure the critical phase of psychotic illness. This result corresponds to those of other studies that indicate that nurses’ presence, caring and interactions with the person in care are important for providing safety and security [20, 21, 23, 27]. Our results concerning how nurses’ confirming mental health care acts helped the participants feel better are partially consistent with Koivisto *et al.’s* [20] study, which showed that nurses expressed an understanding of persons’ needs for care and a sensitivity to their needs for distance and space while empowering them to cope with everyday life [20]. Our results also describe how nurses employed peaceful communication within their relationships with the participants to convey understanding and support for the participants, who had difficulties articulating their care needs. According to Buber [45], to be understood is to be confirmed, and to understand another person is to confirm him/her as a unique person with true expression within a confirming act. Our results show that the nurses responsible for providing care concretely used their ability to reflect upon who the person in care is and consider his/her needs and how to provide the best care. We interpret nurses’ provision of care from the perspective of Buber’s [45] concept of confirmation, akin to taking a bold swing of thought into what another person might wish, feel, perceive and think in an attempt to understand the other person, with doing so leading to the confirming act.

The nurses provided mental health care in a way that led the participants to feel understood. In other words, the confirming mental health care act happened within a mutual understanding between the two of them, with few words and a peaceful manner. The participants described this approach as responsive to their need for care. According to Buber [45], the creation of mutual understanding happens in dialogue. The dialogue between the participant and the nurse was constituted by sensitive caring, gestures, and the use of few words (cf. [45]).

Nurses’ confirming mental health care acts, conveyed through their peaceful approach, demonstrated the nurses’ knowledge of the participants’ needs by allowing them to be critically psychotically ill and by meeting their specific mental health care needs in a sensitive way. This result corresponds to previous research [13, 17, 20] describing the sensitive care that nurses provides for persons suffering from psychotic illness. However, our results show the nurses’ assurance and unconditional mental health care, which implied knowledge and the hope that the participants would get better with the time (cf. [45]). Confirmation occurs between persons in care and nurses through mutual understanding, dialogue and interpersonal relationships and within an on-going movement between distance and closeness between the two partners. These new results are important because they contribute to improve our understanding of how to help psychotically ill persons whose abilities to articulate their needs and enter relationships are limited for a while (cf. [13, 14]). The confirming mental health care seem to contribute to the participants’ ability to feel better and become aware of their needs when nurses help them express and/or articulate what they want and need through confirming mental health care act.

The nurses and physicians strove to decrease the participants’ psychotic symptoms. The participants described this act as being heard, listened to and understood. The discussions among the nurses, physicians, and participants allowed for agreement about how to decrease the participants’ symptoms through medical treatment and care. These results are consistent with those of other studies reporting that involvement in treatment and care planning helps patients feel more in control [23, 25, 38] and involved in a positive process of becoming better [30]. However, other studies have shown that some persons find it difficult to participate in discussions about medical treatment [31], especially when they perceive a paternalistic attitude from the physician when physicians and persons in care disagree [32]. In our study, the disagreements between the nurses/physicians and the participants were described as a confirming mental health care act and as occurring in an atmosphere that permitted exchanges of opinions and a willingness to find solutions and agreements about how medical treatment and care decisions should be made. According to Buber [45], a discussion is a dialogical exchange of understanding that is not intended to change another’s opinion; instead, the aim is to speak and listen with a willingness to understand one another’s perspectives and develop a common understanding. This confirming mental health care act seemed to help increase the participants’ self-respect. This result corresponds with results that describe the importance of patients feeling respected and maintaining their integrity in discussions with professionals [20] if nurses are to develop helping relationships [29] and persons in care become better from psychosis [28]. However, our results clarify the complex nature of creating agreement in shared decision-making processes when the professionals and persons in care do not have a mutual understanding of the situation. Our results reflect the support that nurses gave the participants during disagreements; such moments were important to the development of the discussion, and contributed to the participants’ engagement in the decision-making process.

Together with the nurses, the participants, their family members and local mental health professionals were involved in planning discharge and further support. By allowing the participants to participate in the planning, the nurses confirmed them as valuable persons. Through this confirming mental health care act, the participants engaged in dialogue and partnership with the nurse to make plans for further support and care for themselves and their family members at home. Our results are in line with those of studies describing how professionals’ cooperation with the family members of persons receiving mental health care improved these persons’ relationships with their family [27]. It increased persons’ quality of life [25] and decreased their mental health problems [54]. Furthermore, our results indicate that family members who confirmed the participants through love and concern changed the participants’ perceptions of their family members and themselves as persons who matter to one another. According to Buber [45], simply confirming individuals in their social roles, such as “patient”, reduces their image of themselves as unique persons. Because personal confirmation happens between persons in a dialogue, a common understanding of a “we” can arise. From this perspective, our results show that the joint development of the discharge plan represents a confirming mental health care act arising from a partnership among the nurses, the participant and their family members. It creates a common understanding of the need for further support to maintain the participant’s mental health and to prevent new phases of psychotic illness (cf. [55]). It appears that this confirming mental health care act helps the person receiving care feel better and strengthens her or his self-esteem.

The new knowledge gained from our study indicates that confirmation occurs between the participants and the nurses through reciprocal confirming mental health care acts. These acts appear to help participants feel better in a step-by-step manner. Moreover, as a result of the confirming mental health care acts, the participants become aware of their own needs, their self-respect increases, and their self-esteem as a person who matters to other people is strengthened. According to Buber [45], confirmation is essential to the formation of a person’s self and self-esteem, and it requires the continuous repetition of confirming acts that include mutual understanding and dialogue.

Other studies have described confirming mental health care with a focus on the narrative interviews of persons who have experienced psychotic illness. These studies followed a theoretical perspective that corresponds to elements of person-centred care (cf. [5, 9, 56]). However, our results describe how confirming mental health care acts are experienced by participants with acute psychotic illness, and it appears that confirming acts are important for helping them feel better during care in acute psychiatric wards and may enhance their health. Our results, describing the confirming mental health care that helps persons experiencing psychotic illness to feel better, correspond with studies describing an open verbal dialogue-based approach to persons with acute psychosis and their families receiving psychological therapy [54, 57]. However, our results uncover nurses’ confirming mental health care acts that contain care, such as nurses’ silence, gestures and peaceful communication with persons in care in acute psychiatric wards.

### Methodological considerations and limitations

The trustworthiness of qualitative studies depends on rich and well-saturated data and a valid analysis that demonstrates the connection between the data and the results [48, 53]. To ensure trustworthiness of our results, we performed verification strategies at every step throughout the research process (cf. [53, 58]). The data were verified and confirmed during the data collection by discussing the breadth, depth and nuances of the interviews within our research team. This gave KS the opportunity for self-reflection and self-awareness regarding her own pre-understanding of the topic explored and for modification of subsequent interviews to ensure sufficient data collection. As a team, we discussed the analysis based on critical questions about the chosen focus and the coding strategy and sought to achieve agreement on the constructions of the categories (cf. [48, 58]). The codes and categories were compared in several turns with the whole interview text to ensure that the categories covered data. We paid special attention to the possibility that we may have described the results in an idealized manner, and we sought to balance this possibility through a critical discussion of our interpretation and evaluation of the results.

To facilitate the transferability of our results, the context, the participants, the data collection and the analysis are described carefully. Furthermore, the results are presented via rich descriptions, including quotations from the interviews, to increase the ability of readers to evaluate the transferability of our results to other contexts or groups of persons [48]. Our report adheres to criteria for reporting qualitative research [59]. Aspects of our study results are also consistent with results from previous research [24, 46] and provide new knowledge.

Our results are limited due to the small sample size. However, our aim was not to generalise our findings, but rather to describe and understand the topic explored. The aspects of interpersonal interactions in confirming mental health care should be further explored and described based on narratives to mental health nurses providing care to persons experiencing psychotic illness and by the family members of the persons in care.

## Conclusions

Our results emphasize that confirming mental health care acts provided by nurses in acute psychiatric wards help persons experiencing psychotic illness to feel better. Three confirming mental health approaches were identified: 1) Nurses’ confirming mental health care conveyed through understanding of the participants’ critical illness, peaceful communication and unconditional relationships with the participants helped the participants to endure the critical phase of psychotic illness and to become aware of their own care needs. 2) Nurses’/physicians’ confirming mental health care conveyed through understanding of the participants’ suffering, discussion and interpersonal relationships with the participants helped the participants to experience decreased psychotic symptoms and increased self-respect. 3) Nurses’ confirming mental health care conveyed through understanding of the participants’ vulnerability, dialogue and partnership with the participant helped the participants to feel safe by ensuring that further support would be available at home and strengthened the participants’ self-esteem as persons who mattered to other persons. The nurses’ confirming mental health care is attuned to the participant as a person and to her/his specific, changing needs during psychotic illness. Our results indicate that each confirming approach helps the participants feel better in a step-by-step manner throughout the process of becoming in better health.

Clinical situations involving persons in acute phases of psychotic illness who express changing needs demand special knowledge, sensitivity, skills and responsibilities from nurses beyond the standardized guidelines because each person experiencing psychotic illness is both unique and universal. This means that some experiences are common among people experiencing psychotic illness, while other experiences are genuine and differ from the experiences of others, even others with the same diagnosis. A confirming mental health care act is not the event itself (cf. guidelines) but the attuned interaction between the mental health nurse and the person in care as the caring act can generate an on-going cycle of personal confirmation.

We suggest that nurses seriously consider the participants’ experiential knowledge of confirming mental health care to improve mental health care in acute psychiatric wards. It is necessary for nurses to be present and open to the expressions of the changing needs of persons experiencing psychotic illness and to adjust their care to that persons’ current clinical situation. This openness to the other persons’ expressions creates moments of confirmation that concretely help the persons feel better and thereby support their use of their personal and interpersonal resources in the process of becoming in better health. We also believe that knowledge of confirming mental health care should be included in education and training programmes for nurses meaning learning and training regarding how mental health care confirm the person in care. Such training programs can be provided both at hospitals and in educational settings and supervised by trained mental health nurses. The supervision can also be in cooperation with persons who have experienced psychotic illness themselves and are willing and able to share their knowledge for the education of nurses and increase the quality and competence of mental health nurses.

## Abbreviations

KS: Karina Sebergsen
AN: Astrid Norberg
AGT: Anne-Grethe Talseth
